# Defending against SARS-CoV-2: The T cell perspective

**DOI:** 10.3389/fimmu.2023.1107803

**Published:** 2023-01-27

**Authors:** Patricia Almendro-Vázquez, Rocío Laguna-Goya, Estela Paz-Artal

**Affiliations:** ^1^ Instituto de Investigación Sanitaria Hospital 12 de Octubre (imas12), Madrid, Spain; ^2^ Centro de Investigación Biomédica en Red de Enfermedades Infecciosas (CIBERINFEC), Instituto de Salud Carlos III, Madrid, Spain; ^3^ Department of Immunology, Hospital Universitario 12 de Octubre, Madrid, Spain; ^4^ Department of Immunology, Ophthalmology and ENT, Complutense University School of Medicine, Madrid, Spain

**Keywords:** T cell, SARS-CoV-2, vaccination, adaptive immunity, hybrid immunity

## Abstract

SARS-CoV-2-specific T cell response has been proven essential for viral clearance, COVID-19 outcome and long-term memory. Impaired early T cell-driven immunity leads to a severe form of the disease associated with lymphopenia, hyperinflammation and imbalanced humoral response. Analyses of acute SARS-CoV-2 infection have revealed that mild COVID-19 course is characterized by an early induction of specific T cells within the first 7 days of symptoms, coordinately followed by antibody production for an effective control of viral infection. In contrast, patients who do not develop an early specific cellular response and initiate a humoral immune response with subsequent production of high levels of antibodies, develop severe symptoms. Yet, delayed and persistent bystander CD8+ T cell activation has been also reported in hospitalized patients and could be a driver of lung pathology. Literature supports that long-term maintenance of T cell response appears more stable than antibody titters. Up to date, virus-specific T cell memory has been detected 22 months post-symptom onset, with a predominant IL-2 memory response compared to IFN-γ. Furthermore, T cell responses are conserved against the emerging variants of concern (VoCs) while these variants are mostly able to evade humoral responses. This could be partly explained by the high HLA polymorphism whereby the viral epitope repertoire recognized could differ among individuals, greatly decreasing the likelihood of immune escape. Current COVID-19-vaccination has been shown to elicit Th1-driven spike-specific T cell response, as does natural infection, which provides substantial protection against severe COVID-19 and death. In addition, mucosal vaccination has been reported to induce strong adaptive responses both locally and systemically and to protect against VoCs in animal models. The optimization of vaccine formulations by including a variety of viral regions, innovative adjuvants or diverse administration routes could result in a desirable enhanced cellular response and memory, and help to prevent breakthrough infections. In summary, the increasing evidence highlights the relevance of monitoring SARS-CoV-2-specific cellular immune response, and not only antibody levels, as a correlate for protection after infection and/or vaccination. Moreover, it may help to better identify target populations that could benefit most from booster doses and to personalize vaccination strategies.

## Graphical Abstract

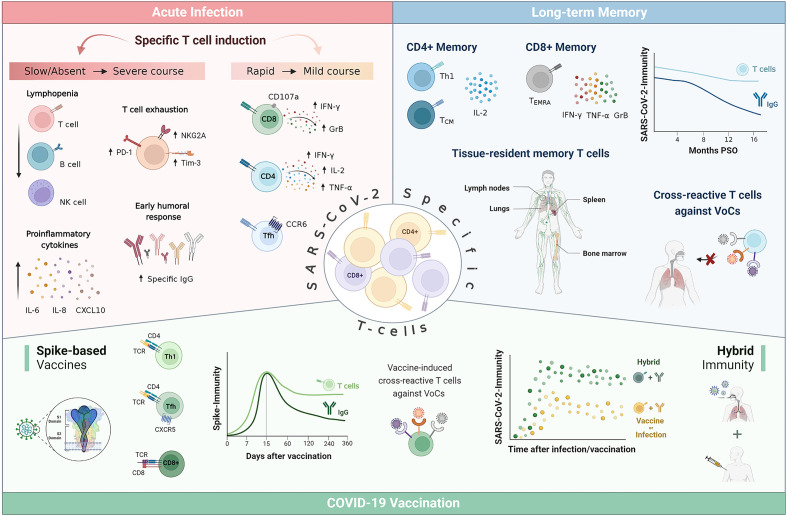

## Introduction

The pandemic caused by severe acute respiratory syndrome-coronavirus-2 (SARS-CoV-2) has resulted in more than 634 million cases of coronavirus disease-19 (COVID-19) and 6.5 million deaths reported worldwide (1). Although the landscape of COVID-19 has evolved substantially since the pandemic onset, understanding how adaptive immune responses develop after infection and vaccination, how this immunity translates into protection against infection and severe forms of the disease, and how we can improve vaccination to reduce transmission remains a major challenge.

Studies on the development of adaptive immunity during the acute infection with other highly transmissible and pathogenic coronaviruses, such as severe acute respiratory syndrome-coronavirus (SARS-CoV) and Middle East respiratory syndrome-coronavirus (MERS-CoV) showed similar dynamics to those reported during the current COVID-19 pandemic (2), which will be discussed in depth later in this review. During SARS-CoV infection, specific serum antibodies are detected 4-7 days after symptom onset (3, 4), and most patients seroconvert within 21 days of onset (5). Similarly, in MERS-CoV infection, the seroconversion process is observed during 15–21 days after infection (6), however, it has been reported that this antibody response is not likely to be correlated with the viral clearance (7, 8). Regarding the T cell response, a dominant Th1 response has been described during SARS-CoV infection (9), whereas in MERS-CoV infection the early increase of CD8+ T cells correlates with disease severity and a dominant Th1 response is observed in the early convalescent stages (10). In addition, a rapid and significant CD4+ and CD8+ T lymphopenia was associated with adverse disease outcome during SARS-CoV (9, 11) and MERS-CoV infection (12), as it has been widely reported during the severe course of COVID-19 (13, 14).

Additionally, studies on the duration of immunity against these coronaviruses report long-lasting SARS-CoV-specific memory T cells up to 17 years after the outbreak of SARS-CoV in 2003 (15) even though antibodies and peripheral memory B cell responses are not detected beyond 3 years after the onset of infection (16, 17). In the same way, T cell responses to MERS-CoV have been detected in patients with undetectable antibody responses up to 2 years after infection (18).

Along these lines, this review summarizes data on the role of SARS-CoV-2-specific T cells in the different phases of natural infection, their relationship to disease outcome and their long-term maintenance. In addition, the impact of vaccine-triggered cell-mediated immunity and the effect of emerging new variants of concern (VoCs) on the ability of T cells to protect from infection and severe COVID-19 are discussed.

## SARS-CoV-2-specific cellular response during acute infection

A coordinated response between innate and adaptive immunity is necessary to control and eliminate SARS-CoV-2 infection. A highly impaired interferon (IFN) type I response, characterized by the absence of IFN-β and low IFN-α production and activity, has been described in severe and critical patients. This defect is associated with persistent blood viral load and exacerbated inflammatory response (19, 20) accompanied by a plasma cytokine signature of elevated CXCL10, interleukin (IL)-6, and IL-8 (21–23). Bastard et al. identified that at least 10% of patients with life threatening COVID-19 pneumonia have neutralizing autoantibodies against type I IFNs (24), while Zhang et al. reported that inborn errors of TLR3- and IRF7-dependent type I immunity underlie critical COVID-19 (25).

Regarding the adaptive immunity, in the context of acute SARS-CoV-2 infection, virus-specific CD8+ T cells with cytolytic capacity against infected cells can be detected as early as day 1 post symptom onset (PSO) (26). Their rapid induction normally occurred within the first 7 days and peaked at 2 weeks PSO (27, 28). This virus-specific CD8+ T cell dynamic has been associated with better COVID-19 outcomes (28–30), as has their capacity to produce high levels of cytotoxic effector molecules, such as IFN-γ, granzyme B and perforin (26, 29, 31). A CD8+ T cell depletion study in non-human primates resulted in a loss of protection in the upper respiratory tract against rechallenge with SARS-CoV-2, which suggests that CD8+ T cells contribute to virological control (32). In addition, Liao et al. demonstrated that a larger proportion of CD8+ T cell effectors with tissue-resident and highly expanded features were present in bronchoalveolar lavage fluid (BALF) from patients with moderate COVID-19 compared to patients with severe/critical infection (33). However, at a local level, lymphocytes with the capacity to kill SARS-CoV-2-infected cells have also been related to further tissue damage and pathogenicity, even late after acute COVID-19. Increased cytotoxic T cells in BALF have been linked to epithelial damage and airway dysfunction (34), and a marked interstitial CD8+ T cell infiltration has been observed in cryotransbronchial biopsies of patients with persistent SARS-CoV-2 pneumonitis (35).

SARS-CoV-2-specific CD4+ T cells can also normally be detected as early as day 2–4 PSO (29, 36) and are more prominent than CD8+ T cell responses (31, 37). Their rapid induction, magnitude and breadth have been associated with accelerated viral clearance and mild COVID-19 (36, 38), whilst their absence within the 22 days PSO has been linked with severe or fatal COVID-19 (39). SARS-CoV-2 infection mainly supports the expansion of Th1 and T follicular helper (Tfh) CD4+ T cells (39–41). Th1 cells have antiviral activity via production of IFN-γ and related cytokines and support cellular and innate immunity against pathogens (42), while Tfh cells are specialized in helping B cells to differentiate into plasmablasts and produce class-switched antibodies within secondary lymphoid organs (43). The majority of SARS-CoV-2-specific CD4+ T cells from COVID-19 patients show a clear IFN-γ, tumor necrosis factor (TNF) and IL-2 protein signature characteristic of canonical Th1 cells (30, 31, 39). While, some studies have reported an incorrect Th polarization with an underrepresentation of Th1 subset in severe COVID-19 patients (44, 45), other authors have informed that a prolonged Th1 cytokine profile was maintained in patients with severe COVID-19 (46). Of note, more recent work has highlighted the importance of Th1-polarized CD4+ T cells to switch towards an IFN-γ and IL-10 functional profile to limit the process of tissue inflammation (47, 48). SARS-CoV-2-specific circulating Tfh (cTfh) cells are generated during acute infection (41, 49) and their frequency has been associated with reduced disease severity (29). A substantial proportion of SARS-CoV-2-specific cTfh are CXCR3-CCR6+ (cTfh17), which has also been observed for the common cold coronavirus, potentially indicative of mucosal airway homing (50). The other two cTfh subtypes, CCR6-CXCR3+ (cTfh1) and CCR6-CXCR3- (cTfh2) have been found to positively correlate with neutralizing activity (50, 51).

The vast majority of SARS-CoV-2 infected individuals seroconvert within 5–15 days PSO (52, 53). Neutralizing antibodies also develop rapidly, on the same time frame as seroconversion (54), reaching maximum levels within 3–5 weeks after infection (55). However, a strong humoral response has been reported in severe COVID-19 patients that is not associated with reduced disease severity (56–59), which had also been observed in MERS-CoV infected patients (60).

## SARS-CoV-2-specific cellular response protects against severe COVID-19

Several studies have revealed that severe COVID-19 patients fail to mount an early, robust T cell response against SARS-CoV-2 and instead initiate a humoral immune response with subsequent production of high antibodies levels unable to efficiently clear the virus (36, 61, 62). Interestingly, results from our group showed that severe patients who subsequently died had a complete lack of specific cellular response while severe patients who survived had a low but detectable number of specific T cells (61). Moreover, we have demonstrated that the specific cellular response against the domain 1 of spike (S1), measured at emergency room is a protective factor against severity (OR per 100 IFN-γ sfu/10^6^ PBMC increment: 0.47, 95%CI 0.20–0.87, p<0.05), independently of age and sex (61). These data suggest that for a total effective control of SARS-CoV-2 infection, an early induction of specific T cells within the first 7 days which peaks at 2 weeks PSO, followed by coordinated antibody production, is necessary (29, 36, 38, 61, 63). The disconnection between humoral and cellular immunity observed in severe patients (29, 36, 62) could be due to a delay in the innate immune response that may lead to a failure in T cell priming (19, 20). Furthermore, other remarkable studies have shown increased frequency of SARS-CoV-2-specific T cells in highly exposed individuals who remain seronegative and PCR negative for SARS-CoV-2 (31, 64–66), suggesting that a rapid deployment of virus-specific cellular immunity suppresses viral replication and successfully aborts infection. In addition, Nelde et al. have shown that the diversity of T cell responses to SARS-CoV-2, not just their magnitude, was associated with mild COVID-19 symptoms, demonstrating that immunity requires recognition of multiple epitopes to control the disease severity (65).

Apart from the lack of early T cell response, severe COVID-19 is also characterized by a marked lymphopenia (13, 14, 67), particularly of CD8+ T cells (68, 69) which resolution correlates with recovery (70). This lymphopenia could partly be due to the robust activated CD8+ T cell recruitment into the lung and brain tissue in critically ill patients (34, 71, 72). However, Szabo et al. have proposed that higher T cell frequencies in the lung correlated positively with survival, whereas higher lung infiltrative myeloid cells correlated with mortality and older age in severe COVID-19 patients (73). Other mechanisms underlying lymphopenia could be an increased T cell apoptosis in patients with COVID-19 (27, 74) or an impaired lymphocyte proliferation (75, 76). However, other studies have described intense T cell proliferation concurring with the lymphopenia (77, 78). Similarly, several studies have reported an exhausted phenotype of CD8+ T cells in severe COVID-19, with an increased expression of inhibitory receptors such as PD-1 and Tim-3 (78–80) and related to the overexpression of the natural killer group 2 member A (NKG2A) in CD8+ T cells (81, 82). On the contrary, recent studies have reported that the expression of exhaustion markers (CTLA-4, PD-1, TIGIT, and Tox) in some SARS-CoV-2-specific CD8+ T cells was not associated with T cell dysfunction (83, 84). An overaggressive CD8+ T cell response or a hyperactive state have also been linked to severe COVID-19 (61, 85, 86).

Aging has also been associated with poor disease outcome, with increased rates of severe and fatal COVID-19 among individuals older than 65 years (29, 87, 88). Age-related remodelling of the immune system, or immunosenescence, is considered the main cause of increased susceptibility to infections, particularly respiratory infections such as influenza, and reduced vaccine effectiveness among the elderly (89, 90). In addition, studies in animal models have reported a decrease in the number of professional antigen presenting cells in the lungs with advanced age (91, 92), which together with the age-associated weaker type I IFN responses may lead to poorer T cell responses to viral infection (93, 94). SARS-CoV-2 capacity to early evade innate immunity (23, 95) may further limit T cell priming in the elderly. Scarcity of naïve T cells in older individuals has also been highlighted as a specific risk factor for severe COVID-19 (96, 97). Finally, the overall disruption of coordinated SARS-CoV-2-specific adaptive responses in older individuals, with a loss of coordination between CD4+ T cell and antibody responses, may contribute to a failure of infection control (29).

In addition, SARS-CoV-2 T cell cross-reactivity, i.e., the existence of memory T cells induced by previous pathogens, could play an important role in the COVID-19 outcome (98). It has been reported that recently seasonal coronavirus infected individuals develop less severe COVID-19 (99). Similarly, pre-existing cross-reactive T cells facilitated the expansion of SARS-CoV-2-specific CD8+ and CD4+ T cell responses during infection (100, 101) and were associated with control of viral replication and abortive infection (66, 102). Although some studies have reported the presence of SARS-CoV-2 cross-reactive T cells in 20% to 80% of unexposed individuals (15, 37, 103, 104), in other series no cross-reactive T cells have been found (30, 105). However, these disparities may be due to the different technical approaches performed (106). T cell cross-reactivity has been described to be more frequent among children and young adults (107, 108), and to be mainly directed against the domain 2 of the SARS-CoV-2 spike protein due to its moderate amino acid conservation (107, 109).

## Duration of T cell immunity and its potential role in protection against re-infection

Numerous studies have assessed the duration and characteristics of both cellular and humoral immunity following SARS-CoV-2 infection. To date, the presence of SARS-CoV-2-specific memory T cells in blood has been demonstrated 10 (110), 12 (111) and up to 22 months (112) post-infection. Likewise, SARS-CoV-2-specific antibodies have been detected 12 (113), 13 (114) or 15 months (115) after the onset of infection. Nevertheless, the kinetics of both branches of adaptive immunity exhibit different patterns, as cellular response tends to remain stable (61, 116–118) while antibodies decay rapidly in early convalescence (119–121).

It has been described that SARS-CoV-2 memory response is skewed towards more CD4+ helper T cells than CD8+ cytotoxic T cells (37, 116). More specifically, Cohen et al. have described that the median frequency of SARS-CoV-2-specific memory CD4+ T cells was 0.51% of the T cell repertoire, while the median frequency of SARS-CoV-2-specific CD8+ T cells was 0.2% (122). Nevertheless, a wide range in magnitude of specific T cells among the analysed patients has been observed probably due to the differential kinetics of expansion and contraction of the two cell populations in convalescent individuals (123). Most of the virus-specific memory CD8+ T cells are terminally differentiated effector memory cells (T_EMRA_) (116, 124), antigen-experienced cells that re-express the naïve cell marker CD45RA (125), and which in the context of influenza have been associated with protection against severe disease (126). These SARS-CoV-2-specific CD8+ memory T cells secrete mainly IFN-γ, TNF-α, and granzyme B (122). CD4+ T cell memory is predominantly T central memory (T_CM_) with phenotypic features characteristic of Th1 polarization, lymph node homing, robust helper function and longevity (116, 118, 124). The dominant cytokine response in CD4+ T cell subsets is IL-2, indicating proliferative potential, which may suggest a robust long-term immune memory (61, 118).

Apart from circulating memory T cells, tissue-resident SARS-CoV-2-specific memory T cells have also been reported particularly in the airway and associated lymph nodes, but also in oropharyngeal tonsils, bone marrow and spleen, and their frequency correlated with that of circulating specific T cells (127, 128). A study in convalescent patients found tissue-resident SARS-CoV-2-specific CD8+ T cells in nasal mucosa, which had overlapping T cell receptors with circulating T cells within each patient, and suggested that these local memory cells could rapidly attenuate re-infection by SARS-CoV-2 (129).

In addition to the long-term maintenance of specific T cell responses and their capacity to prevent severe disease (29, 36, 38, 61, 63), an increasing number of studies have demonstrated that infection-induced SARS-CoV-2-specific T cells largely tolerate the amino acid mutations of the different VoCs. The impact of the different mutations in the early VoCs (Alpha, Beta, Gamma, and Epsilon) is limited therefore the majority of CD4+ and CD8+ T cell responses are preserved (130–132). Studies on the impact of newer variants such as Omicron, which holds more than 50 mutations, 37 of them in the spike protein (133), have reported that 70% to 90% of the CD4+ and CD8+ T cell response to spike was maintained (134, 135). The reduced likelihood of cellular immune escape of new SARS-CoV-2 variants is probably partly related to the high polymorphism of the HLA system. Therefore, the set of viral epitopes recognized by each individual´s lymphocytes may be different, and overall, at the population level, be broader (136–138). The progressive emergence of VoCs that mainly accumulate mutations in the spike protein (139, 140) and thus have an increased ability to evade neutralizing antibodies (141–144) has led new analyses to focus on determining correlates of protection against severe disease rather than infection (98). Epidemiological observations have reported that despite the increasing infection rate related with the successive waves of VoCs (145, 146), protection against hospitalization and death due to re-infection have remained extremely high (147, 148). Given the waning humoral immunity after infection (119, 121) and the VoCs ability to evade neutralizing antibodies (141, 142, 144), it may be suggested that T cells play an important role in this protection against severe COVID-19, also in the context of VoC infection.

## T cell response after vaccination

Studies on the immunodominant regions of SARS-CoV-2 have been particularly important for vaccine development. Griffoni et al. have reported that T cells can recognize up to 10 different SARS-CoV-2 proteins, with spike being the most immunodominant, followed by nucleocapsid, membrane, and other non-structural proteins (149, 150). These data, together with the fact that the receptor binding domain (RBD) of the SARS-CoV-2 spike protein is the main target of neutralizing antibodies (53, 151), led to the development of different spike-based vaccine candidates.

Up to date, ten COVID-19 vaccines have been approved by the World Health Organization (WHO) for global use. Among them, four different vaccination platforms can be distinguished: messenger RNA (mRNA) vaccines (Moderna’s Spikevax mRNA-1273 and Pfizer-BioNTech’s Comirnaty BNT162b2), adenovirus vector-based vaccines (AstraZeneca’s Vaxzevria, Covishield ChAdOx1 and Johnson & Johnson-Janssen’s Ad26.COV2.S), inactivated virus vaccines (Sinopharm’s Covilo, Sinovac’s CoronaVac, and Bharat Biotech’s Covaxin) and adjuvanted protein vaccines (Novavax’s Nuvaxovid and Covovax NVX-CoV2373) (139, 152). All have demonstrated high rates of efficacy in preventing COVID-19, especially protecting against severe disease and death (153–158). Since vaccination protocols started in December 2020 with BNT162b2 vaccine, it has been estimated that global anti-SARS-CoV-2 vaccination saved nearly 20 million lives in its first year of application (159).

Several studies have shown that mRNA vaccines elicit an early and potent humoral immune response (160, 161) that declines sharply beyond 3 months post-vaccination (162–165). Neutralizing antibody titers developed after mRNA SARS-CoV-2 vaccination exhibit similar dynamics (112, 166, 167), with a half-life of approximately 60 days (168). In contrast, it has been reported that the Ad26.COV2.S vaccine induces lower initial levels of neutralizing antibodies but these remain stable up to 8 months after vaccination (166, 169).

It has also been proven that COVID-19 vaccination elicits a robust T cell response. As expected, mRNA- and adenovirus vector-based spike vaccines induce only spike-specific T cells (112, 161, 170–172), whereas inactivated virus vaccines induce T cells capable of recognizing several SARS-CoV-2 antigens (173, 174). As in natural infection, the phenotypic profile of mRNA vaccine-induced T cells is predominantly Th1 (61, 172, 175–177). In addition, induction of follicular helper CD4+ T cells along with cytotoxic CD8+ T cells has been described (132, 178, 179). Early induction of these specific CD8+ T cells may explain the vaccine-mediated protection against severe disease that has been described as early as ten days after prime vaccination (180), when neutralizing antibodies are barely detectable (176, 181). Similarly, studies in non-human primates highlight the potential of vaccine-induced CD8+ T cell responses to contribute to viral load reduction and COVID-19 containment (182–184). In addition, it has also been demonstrated that vaccine-induced T cells are able to cross-recognize VoCs (185–189), while VoCs partially evade neutralizing antibodies elicited by vaccination (190–192), as it happens in natural infection. In particular, vaccine-induced T cells show high cross-reactivity (over 80%) against Omicron (135, 193, 194), even in the absence of high neutralizing antibodies titers (195, 196). Vaccine induced T cell memory has generally been described as long-lasting as and more stable than humoral response even though it usually shows some degree of contraction within the first 3 months after vaccination (112, 139, 185, 197).

Comparisons of T cell immunogenicity and memory elicited by the different COVID-19 vaccines platforms have been challenging and limited, mainly due to the lack of standardized cellular assays, unlike the standardization of binding and neutralizing antibodies provided by WHO (198). Comparative studies on the development and maintenance of immunity elicited by different vaccine platforms highlight that mRNA vaccines induced the highest CD4+ T cell responses (199, 200). In addition, both the CD4+ T cell peak and memory responses developed by the BNT162b2 vaccine were lower than those generated by the mRNA-1273 vaccine (200, 201). The acute and memory responses of CD8+ T cells generated by BNT162b2 were comparable to those of mRNA-1273, but slightly lower in frequency and multifunctionality (200). Timing and vaccine dosing regimens, as mRNA-1273 contains 100 μg mRNA, while BNT162b2 contains 30 μg (200), may be behind these differences. CD8+ T cell responses were particularly high after Ad26.COV2.S vaccination (166, 199) and low, but detectable, after NVX-CoV2373 immunization (200). This variable immunogenicity among the COVID-19 vaccines could explain the differences in the efficacy and effectiveness that have been reported (157, 180, 202, 203). In all cases, vaccine efficacy against hospitalization remains stable over time, unlike efficacy against infection (204, 205). This suggests that, as in natural infection, vaccine-induced T cells are contributing to the control of COVID-19, preventing the development of severe disease, in a context of waning humoral immunity, independently of the SARS-CoV-2 variant evolution.

## Hybrid immunity against SARS-CoV-2

Despite protection against severe COVID-19 and death has been achieved, an increase in infection rates has been observed (145, 146, 206), probably due to the combination of the successive emerging VoCs and a decreasing immunity landscape. Seroprevalence surveys suggest that more than half of the global population has been infected with SARS-CoV-2 (148). This, together with the massive application of vaccines, has triggered a great interest in the characterization of the so-called hybrid immunity against SARS-CoV-2. The term hybrid immunity applies to individuals with a previous SARS-CoV-2 infection who were subsequently vaccinated against SARS-CoV-2 or vice versa, thus exhibiting a combination of infection- and vaccine-induced immunity (96, 207). Numerous studies have revealed that individuals with previous SARS-CoV-2 infection develop unusually strong immune responses to COVID-19 vaccines (208, 209). Specifically, the neutralizing antibody titers were dramatically higher in previously infected individuals receiving at least one dose of COVID-19 vaccine (161, 207, 210) and their memory B cells were increased 5- to 10-fold compared with natural infection or vaccination alone (161, 211). Similarly, multiple studies have observed an overall increase in T cell response in hybrid immunity compared to either infection or vaccination (112, 212, 213). It has been shown that COVID-19 vaccines can substantially boost spike-specific CD4+ T cell responses, and have a modest effect boosting spike-specific CD8+ T cells, in previously infected individuals after immunization (98, 185, 211). Likewise, hybrid immunity resulting from vaccination plus subsequent infection (breakthrough infection) also results in equally robust adaptive immune responses (214, 215).

It should be noted that most COVID-19 vaccines in use consist of a single antigen, the spike protein, whereas 29 different viral proteins are present in SARS-CoV-2 (216). Therefore, the breadth of the resulting immune response following COVID-19 vaccination is more restricted than in natural infection (207, 217). One of the advantages of hybrid immunity is that it consists of both spike and non-spike memory, resulting in a broader repertoire of virus-specific antibodies (218, 219), B cells (217, 220) and T cells (83, 217). Comparative analyses have reported that hybrid immunity provides greater and more durable protection against re-infection than immunity triggered by two or three doses of COVID-19 vaccine in uninfected individuals (221, 222) and this superior protection conferred by hybrid immunity has been maintained even against VoCs, including Omicron (218, 219, 223, 224). However, even though additional antigen exposure from natural infection substantially boosts the quantity and breadth of immune response, recent studies have shown no differences between T cell cross-reactivity against Omicron in vaccinated- compared to hybrid immune-subjects (188, 225).

It has also been shown that infection following spike-based vaccination does not prevent the development of a non-spike-specific T cell response, but on the contrary, it gives rise to a broad repertoire of T cells specific against other SARS-CoV-2 antigens (83, 112). This indicates that individuals with breakthrough infection do not have a preferential bias toward spike responses, which is especially important given the continued emergence of VoCs and the current increase in the rate of breakthrough infection (131, 226).

One of the most recently discussed concerns has been the possible deleterious effect of the repeated SARS-CoV-2 antigen exposure on the protective capacity of memory T cells. Repeated flu vaccination has been shown to result in a ‘vaccine exhaustion’ with significantly reduced protection rate (227). However, and regarding cellular immunity in particular, recent work by Minervina et al. has shown that repeated antigen exposure did not result in SARS-CoV-2-specific T cell dysfunction (83). Despite the expression of T cell exhaustion markers (CTLA-4, PD-1, TIGIT and Tox), SARS-CoV-2-specific CD8+ T cells retained their proliferative capacity. Based on these evidences future immunization boosters with vaccine platforms including a variety of SARS-CoV-2 antigens seems to be recommendable.

New COVID-19 vaccines are being developed to improve efficacy and protection against breakthrough infections, especially relevant in the current situation with periodic emergence of new VoCs. In this regard, new mRNA vaccine platforms that contain equal amounts of mRNAs encoding the ancestral SARS-CoV-2 and Beta or Omicron variant spike proteins have been developed (228–230). These bivalent boosters enhance the humoral immune response, eliciting superior and more durable neutralizing antibody responses against VoCs compared to monovalent vaccine formulations (229, 231–233). However, the effect of bivalent vaccines on T cell immunity remains less understood and further studies are still required. A recent study by Tan et al. has shown that bivalent booster vaccination leads to a robust recall of memory T cell responses within the first 7 days after boost, that exhibit cross-reactivity against Omicron BA.1 and BA.5 variants regardless of the priming regime administered (234).

Another interesting new strategy for the vaccination against COVID-19 is the application of intranasal booster vaccination, as mucosal immunity has been shown to protect against breakthrough infections (128, 129). Currently approved intramuscular COVID-19 vaccines induce variable levels of mucosal humoral immunity (235–237), and while a recent study reported the presence of high frequency of spike-specific T cells in the airways of mRNA-vaccinated individuals (238), others detected spike-specific T cells only in the BALF of SARS-CoV-2 convalescent patients but not in the BALF of vaccinated-only individuals (239, 240). On the contrary, mucosal vaccination can elicit strong adaptive responses both locally and systemically as reported in animal models. It has been shown in mice that intranasal vaccination against SARS-CoV-2 led to the induction of broadly neutralizing antibodies and polyfunctional central memory T cells locally, in the draining lymphoid organs, and systemically in the spleen (241). Mao et al. propose the use of intranasal booster administration which can induce robust tissue-resident memory B and T cell responses, as well as boost systemic immunity (242). Importantly, an intranasal vaccine with non-spike antigens in *Cynomolgus macaques* reduced the viral load two days after being challenged by intranasal SARS-CoV-2. The viral control was observed in the absence of neutralizing antibodies, and it was linked to the induction of specific CD8+ T cell responses (183). Finally, intranasal immunization has been shown to provide protection against VoCs in animal models (243, 244). These data highlight both the protective value of SARS-CoV-2-specific T cells resident in the upper respiratory tract and the effective next-generation COVID-19 intranasal vaccine approach to induce mucosal immunity against current and future VoCs, which could be a promising strategy to reduce breakthrough infections and curb SARS-CoV-2 transmission.

## Concluding remarks

Much information has been generated since the onset of the COVID-19 pandemic about the role of T cell-mediated immunity in SARS-CoV-2 infection and its association with disease control. It has been shown that, although neutralizing antibodies play a dominant role in the prevention of infection, an adequate disease control depends on the synchronized function of the two branches of the adaptive immunity, where the coordinated development of an early T cell response followed by a controlled humoral response is essential.

Although the development and massive application of COVID-19 vaccines has led to a dramatic decrease in the frequency of severe disease and death, it is still necessary to establish accurate correlates of protection. Thus, increasing evidence supports that since antibody titers are not a surrogate indicator of the magnitude of memory T cells, the use of antibody serodiagnostic tests alone will not be a robust indicator of protective immunity against SARS-CoV-2. In addition, it has been shown that T cells remain largely stable over time while antibodies and their neutralizing activity sharply decrease. This fact, together with the robust evidence that the T cell response is preserved against VoCs, as opposed to neutralization, further highlights the association between the cellular immunity and the decreased rates of severe disease, regardless of SARS-CoV-2 variant evolution. Along these lines, it would be essential to implement a cellular response surveillance program to fully understand the extent of immunity among the population and to be able to identify vulnerable groups.

In parallel, comparative studies between protection conferred by natural infection, vaccination or hybrid immunity have highlighted the value of developing a broad immune repertoire against SARS-CoV-2. Therefore, the inclusion of viral antigens other than spike in new vaccine designs could result in further benefit.

## Author contributions

All authors contributed to conception, writing, and editing of the final manuscript. PA-V and RL-G contributed equally to literature review and first draft. All authors contributed to the article and approved the submitted version.
